# Associating expression and genomic data using co-occurrence measures

**DOI:** 10.1186/s13062-019-0240-2

**Published:** 2019-05-09

**Authors:** Maarten Larmuseau, Lieven P. C. Verbeke, Kathleen Marchal

**Affiliations:** 1 Department of Information Technology, Ghent University – Imec, Technologiepark-Zwijnaarde 126, 9052 Ghent, Belgium; 2 Department of Plant Biotechnology and Bioinformatics, Ghent University – Imec, Technologiepark-Zwijnaarde 126, 9052 Ghent, Belgium

**Keywords:** Expression data, Co-expression, Data integration, Breast cancer

## Abstract

**Abstract:**

Recent technological evolutions have led to an exponential increase in data in all the omics fields. It is expected that integration of these different data sources, will drastically enhance our knowledge of the biological mechanisms behind genomic diseases such as cancer. However, the integration of different omics data still remains a challenge. In this work we propose an intuitive workflow for the integrative analysis of expression, mutation and copy number data taken from the METABRIC study on breast cancer. First, we present evidence that the expression profile of many important breast cancer genes consists of two modes or ‘regimes’, which contain important clinical information. Then, we show how the co-occurrence of these expression regimes can be used as an association measure between genes and validate our findings on the TCGA-BRCA study. Finally, we demonstrate how these co-occurrence measures can also be applied to link expression regimes to genomic aberrations, providing a more complete, integrative view on breast cancer. As a case study, an integrative analysis of the identified *MLPH*-*FOXA1* association is performed, illustrating that the obtained expression associations are intimately linked to the underlying genomic changes.

**Reviewers:**

This article was reviewed by Dirk Walther, Francisco Garcia and Isabel Nepomuceno.

**Electronic supplementary material:**

The online version of this article (10.1186/s13062-019-0240-2) contains supplementary material, which is available to authorized users.

## Background

Systems genetics approaches that collect genomic information with matching transcript information from phenotypically well characterized individuals provide a powerful way to study the molecular mechanisms underlying complex phenotypes. For this reason systems genetics approaches have become increasingly popular in the domain of cancer genomics [1–3]. However, the analysis and integration of these different data sources is non-trivial. Indeed, although many integrative or multi-omics models have been proposed [4–6], the relation between genetic variants and subsequent changes in gene expression remains poorly understood [7, 8]. A fundamental problem when integrating expression data with genomic information lies in the different nature of both datasets. While expression data is quantitative, consisting of continuous values that indicate the degree to which a gene in a sample is being transcribed, genomic data is essentially qualitative. A common way to deal with this problem is to convert the continuous expression measurements into more qualitative, discrete values. Two strategies exist for this conversion: the identification of a set of differentially expressed genes [5] and the direct binning of expression data into discrete categories [6]. The focus of this work will be on the second strategy, where expression data is binned or discretized as a preprocessing step. This discretization is non-trivial and many different techniques exist, as reviewed in the work of Gallo et al. [9] in the context of single-source expression analysis. The advantages of discretization in single-source expression analysis are mainly related to mathematical convenience [10] and reduction of noise in the data [11–13]. However, in the context of data integration for cancer research there are some other arguments for the discretization of expression data.

First, many continuous methods rely on mutual information or correlation based measures to define whether the expression of two genes is related or not [14]. While these measures are suited to describe co-expression, they might not be the measure of choice, if the expression of two genes is related only under a specific set of conditions. For cancer this might be a very relevant concern as a cohort of samples often contains several subtypes and/or network rewiring due to genomic changes that affect local gene expression behavior [15, 16]. The reconstruction of these condition-specific modules or subnetworks from expression data has been a research question for over 20 years [17–19] and some methods indeed rely on data discretization as a preprocessing step [20]. The problem finding condition-specific modules is in essence a bi-clustering problem, where one tries to find modules of genes that show similar behavior in a subset of the samples [21, 22].

Second, the use of correlations for the identification of co-expressed genes assumes that an increase in one gene (e.g. transcription factor) will trigger a proportional increase or decrease in another gene (target) [23]. However, this assumption is often a simplification of reality as many examples of complex feedback mechanisms in the human body exist, where definite changes only take place once a certain threshold is crossed [24, 25]. It therefore makes sense to describe the expression of a gene in terms of discrete regimes and to model the gene interaction network as a complex nonlinear system, consisting of many discrete states. These networks are perturbed by an external trigger, such as the occurrence of a somatic mutation, that affects many genes in the network and causes them to undergo a shift in expression.

In this work we want to show that the discretization of expression data into expression ‘regimes’ also yields an important third benefit, related specifically to the integration of expression data with genomic information. The discretization of expression data essentially converts the quantitative transcriptome measurements into qualitative data, that indicate in which expression regime a gene is found to be. Because genomic information is in essence qualitative, integration of transcriptome and genomic data sources can now be done by simply counting how many times a given genomic aberration co-occurs with an expression regime. In the same way, phenotypes and clinical subgroups can be analyzed by counting how many times they co-occur with aberrations and/or regimes, and identifying the most overrepresented aberrations and regimes using a suitable association measure. To this end, we propose the use of ‘co-occurrence measures’ that are calculated between subgroups of a cohort, rather than over the whole cohort.

In this work we present evidence that the expression profile of many important genes in breast cancer, actually follows a bimodal distribution, where the mode or ‘regime’ of a gene contains important clinical information. The presence of these regimes allows for a drastic simplification of the subsequent analysis, as expression data can be discretized in a biologically sound way without much loss in information content. Using a breast cancer dataset, we demonstrate that measures that count the co-occurrence of these expression regimes between different genes (‘co-occurrence measures’) are a suitable association measure for the analysis of expression data. We compare the co-occurrence measures with two commonly used measures, the Pearson correlation coefficient and Mutual Information [26]. The genetic associations obtained when using these co-occurrence measures seem to closely reflect the underlying genomic changes and are complementary to what is found using the conventional association measures. Finally, we demonstrate how these co-occurrence measures can also be used to associate mutation and copy number information with expression regimes, obtaining a more complete, integrative picture of the different elements that constitute a particular phenotype or clinical subgroup. As a case study we analyze the relation between *MLPH* and *FOXA1* in breast cancer.

## Results

**Fig. 1:**
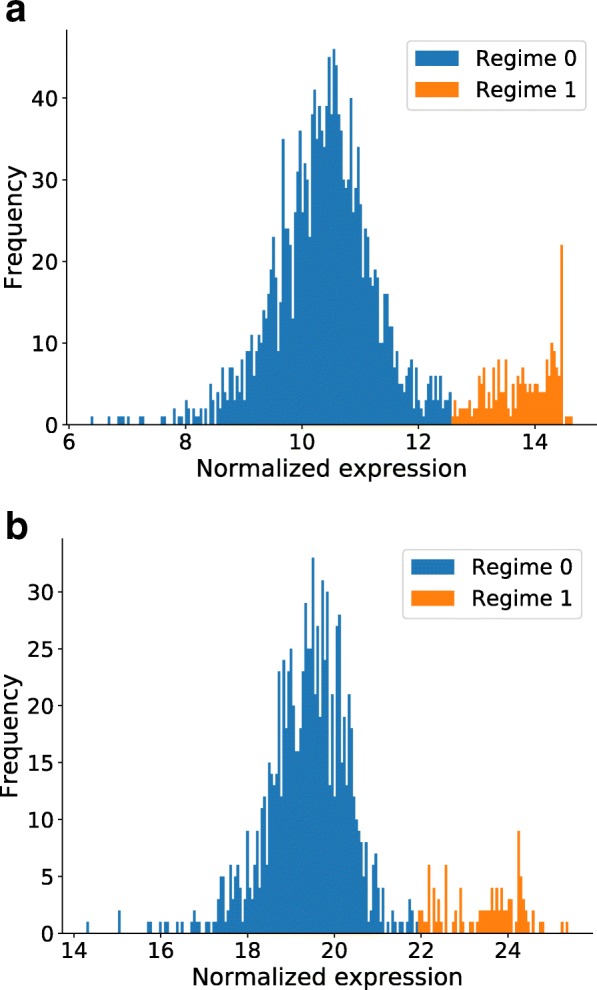
The expression profile for the notorious breast cancer gene ERBB2/HER2 across the METABRIC (**a**) and TCGA (**b**) cohort. In both profiles the expression distribution over the samples approximately follows a bimodal distribution. The expression data is discretized by assigning a 0 to the samples belonging to the blue distribution and a 1 to the samples in orange

Many clinically important genes in breast cancer, e.g. ESR1 [27] and ERBB2 [28], appear to follow a bimodal expression distribution across the samples of a cohort. We hypothesized that these regimes reflected the underlying genomic changes. For instance, in the case of ERBB2, a gene that is known to be amplified in breast cancer [28], we could observe that many samples in regime 1 indeed have an amplification in ERBB2. To be able to perform a large-scale analysis of these expression regimes, we used a Gaussian Mixture Model (GMM) to assign samples to a cluster/regime (see Fig. 1) and repeated this procedure for every gene in the METABRIC [29, 30] study (see methods). Because there is no a priori reason why expression profile would bimodal, we select for each gene the number of regimes based on the Bayesian Information Criterion (BIC) [31]. The only parameter in the discretization procedure is the maximum number of regimes that a gene profile can consist of. Using this approach we verified whether the genes from the KEGG pathway [32] associated with breast cancer showed this multimodal behavior (see methods). We observed that all genes in the breast cancer pathway indeed displayed this multimodal behavior. In total, we found that about 60% of the genes in the METABRIC study (114,652 out of 24,630 different transcripts measured), displayed a multimodal behavior. In this work, we show that these modes or ‘regimes’ of a gene actually convey important clinical information and can be associated to underlying genomic changes.

### Information content of expression regimes

To estimate how much information is lost during the discretization step, Random Forest classifiers are trained on the METABRIC dataset to predict the PAM50 subtypes, including the Claudin-low and Normal-like subtypes [33–35]. The first classifier is trained on the original, continuous data and compared to classifiers trained on the GMM discretized data, again comparing the three values for the maximum number of regimes. Random Forest classifiers are chosen, because the underlying decision trees also threshold the data, but in a sequential and supervised fashion. Consequently, the classification performance reflects how the unsupervised discretization of the expression data compares to a supervised thresholding. We also compare to a naive binarization strategy, in which the expression is normalized to unit variance and zero mean and then binned into three categories (]-∞, − 1.5σ[, [− 1.5σ, 1.5σ],]1.5σ, ∞[; where σ is the standard deviation).

**Fig. 2:**
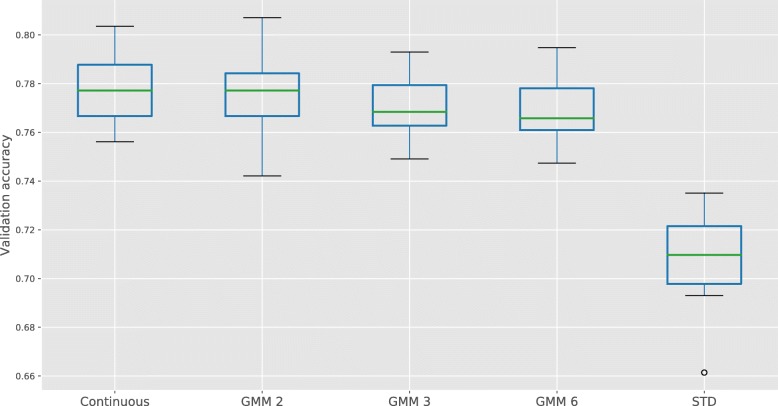
Accuracy on the validation set for PAM50 subtype classification, comparing training on continuous microarray data to training on GMM discretized data and a naive discretization strategy (STD). For the GMM discretization, we allowed the maximum number of regimes to be 2 (GMM 2), 3 (GMM 3) and 6 (GMM 6). Cross-validation was repeated 20 times, with a 0.7/0.3 training/validation ratio using a Random Forest classifier with 1500 trees [49]

Figure 2 shows a Boxplot of the validation accuracy when training classifiers to predict the PAM50 subtype. It shows that the validation accuracy on the GMM discretized data is only slightly worse than the continuous data, in line with what was found in Ding et al. [12]. Conversely, a naive discretization strategy leads to a worse performance. Surprisingly, the model trained on the binary data achieves a slightly higher average accuracy, at the cost of a larger standard deviation, compared to models trained on data that consist of more regimes (GMM 3 and GMM 6). In the remainder of this work we will limit to the number of regimes to 2, resulting in binary expression data, and show that this still allows to find many important associations between gene expression and the underlying genomic alterations. For other cancer types, this assumption may not hold, but the presented framework (and accompanying implementation) supports the general case of *n* regimes. In the remainder of this work, we will use the convention that regime ‘0’ denotes the low expression regime of a gene and ‘1’ the high expression regime, in accordance with Fig. 1.

### Clinical relevance of the expression regimes

The results in Fig. 2 indicate that, for the PAM50 classification, not much information is lost in the GMM discretization step, even if we restrict the discretization to only two values (the GMM 2 scenario). However, if these binary expression regimes are truly informative, they should also convey important information about the prognosis. For each gene that has two different expression regimes in the METABRIC study, we calculated the age-corrected hazard ratio between samples that are in regime ‘0’ and ‘1’. Table 1 shows the 5 genes that have the highest hazard ratio, as well as the regime (‘0’/‘1‘) and the number of samples in which this regime is present (see methods). Here the hazard ratio of 2.31 for *SPATA4* implies that samples where *SPATA4* is in regime ‘1’ decease at a 2.31 faster rate than the group where *SPATA4* is in regime ‘0’.

**Table 1:** Top 5 genes with the highest hazard ratio

	Gene	Hazard ratio	N samples	Regime
1	SPATA4	2.31	306	0
2	UCP1	2.24	159	0
3	AURKA	2.17	796	1
4	GLA	2.14	243	0
5	PGAP3	2.12	238	0

**Fig. 3:**
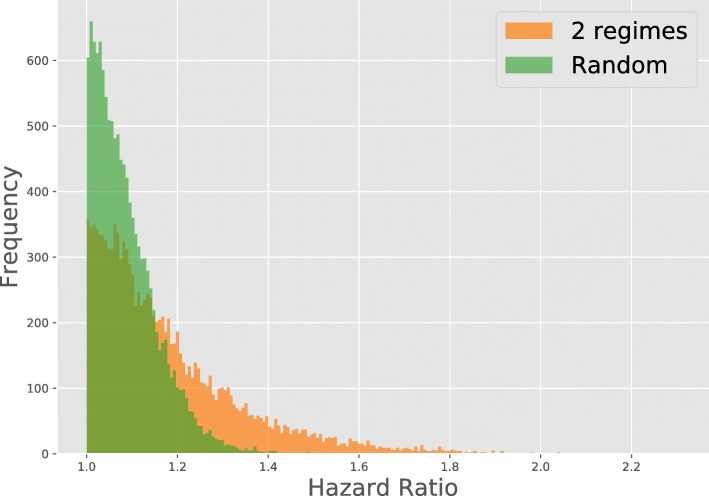
Comparison of the Hazard ratio for the data that was binarized in 2 regimes (orange) and the same binarized data where the samples were randomly permuted for each gene (green)

Out of these five genes, only *SPATA4* has not been reported in breast cancer literature [36–39]. Figure 3 shows the distribution of hazard ratios, comparing to a random permutation of samples across each gene, illustrating that the hazard ratios found are not generated by chance.

### Co-occurrence of expression regimes as an association measure

**Fig. 4:**
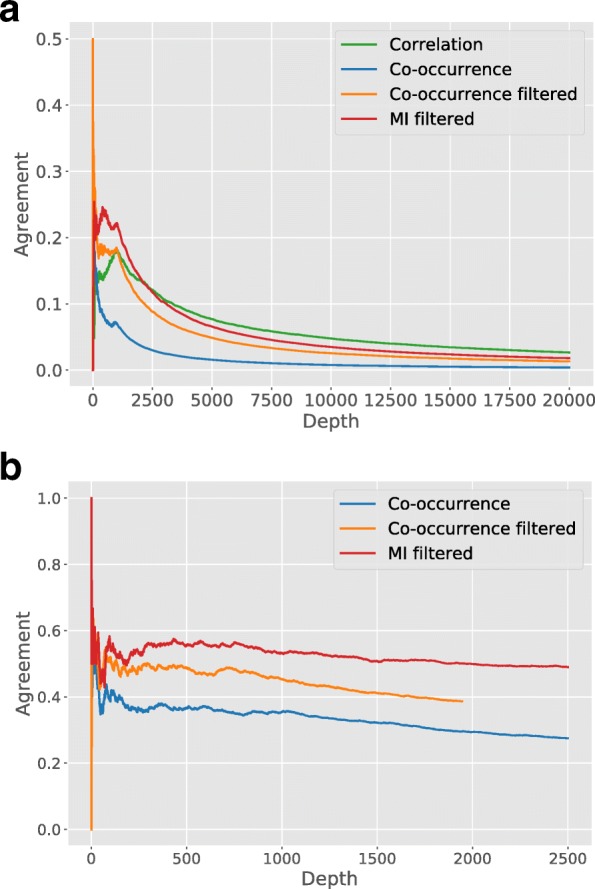
**a** Agreement between the associations found on TCGA-BRCA with METABRIC, for three different assocation measures (see text). **b** The agreement of the TCGA and METABRIC associations between expression regimes and mutations

Our previous results seem to suggest the existence of clinically relevant regimes in the expression profile of a gene. A perturbation, e.g. a genomic aberration, can trigger a regime shift. Even if the trigger of this regime shift is unknown, it is still possible to identify pairs of genes that often co-occur in their regimes. To find such pairs of genes, the *p*-value under the assumption of independence is calculated between all genes (see methods). Unsurprisingly, many pairs of genes are found that co-occur significantly more than expected by random. 1,342,459 gene pairs are significant at a conservatively corrected 0.001 level (see methods), illustrating that the expression of genes is tightly connected and that co-occurrence of these regimes can indeed be used as an association measure. To get an idea of how consistent this association measure is, we compare the associations to those found using Pearson correlation coefficient on the continuous data and Mutual Information (MI) on the discretized data (see methods). In addition, we compared all associations found on the METABRIC to another breast cancer study (TCGA-BRCA, see methods). For both studies we thus obtain 3 ranked lists of associations, one for each association measure. Each line in Fig. 4a is obtained by simultaneously going down the TCGA-BRCA and METABRIC ranked lists for the same measure, at each depth calculating the relative overlap or agreement between the two lists [40]. In case of two identical lists, the result would be a perfect agreement of 1, for each depth. The small peak at depth 2 for the co-occurrence measure is due to the strong association between *MLPH* and *FOXA1*, that is found in both datasets (cf. infra).

Note that the measures that rely on discretized data, i.e. the co-occurrence measure and the MI, are at a disadvantage. Indeed, if a gene is multimodal in one dataset and unimodal in the other dataset, then all associations found in the first dataset cannot be found back in the second dataset. To be able to compare the performance of the association measures, both datasets are filtered, keeping only genes that are bi-modal in both datasets. The impact of this can be seen in Fig. 3a by comparing the blue line (co-occurrence on the unfiltered datasets) to the orange line (Co-occurrence filtered). On the filtered datasets it can be seen that both MI and co-occurrence lead to higher consistency between the top ranked associations. At larger depth the correlation measure is slightly more consistent, but note that the two datasets show a rather poor overlap for each of the measures. Possible reasons for this poor overlap are the differences in technology and cohort composition (see methods).

Interestingly, we note that the overlap between the associations is rather small for the different methods (see Additional file 1: Figure S1), indicating that the different association measures provide complementary information.

Among the most significant gene pairs we see that many genes occur several times, such that small cliques of co-occurring genes are formed (see Additional file 2: Figure S2 and Additional file 6: Table S1). An important point is that these subnetworks are in fact bi-clusters, as the samples in which these regimes are present together are also known.

### Data integration using co-occurrence

By discretizing the expression data, we have essentially converted the quantitative measurements into qualitative data, indicating the regime to which a gene belongs. Based on the results from the previous section, measures such as MI and co-occurrence can be used to identify associations between this qualitative data. Because genomic information is mostly qualitative, these measures can also be applied to calculate the co-occurrence or MI between mutations and expression. Here too, we assessed how well the associations in METABRIC agree with TCGA. The results are shown in Fig. 4b and can be compared to Fig. 4a. It can be seen that associations between mutation and expression data are more consistent between the two datasets, for both the MI and the co-occurrence. Because the co-occurrence measure used here represents a *p*-value, pairs can be selected based on a chosen significance level. Aiming for a significance level of 0.001 and taking into account multiple hypothesis testing (see methods), we obtain 1876 significant pairs for METABRIC and 802 for TCGA. The discrepancy in number of significant pairs is due to the different sample size for the two datasets. In general we see that a higher significance level does indeed lead to a better overlap between the datasets (see Additional file 3: Figure S3). Also, we observe that from the top 1000 associations, 682 overlap between MI and Co-occurrence, which more than what was found for expression (see Additional file 1: Figure S1). More than 97% of the associations involve mutations in *TP53* in both studies, where *TP53* is the strongest associated with low expression in *ESR1*.

**Fig. 5:**
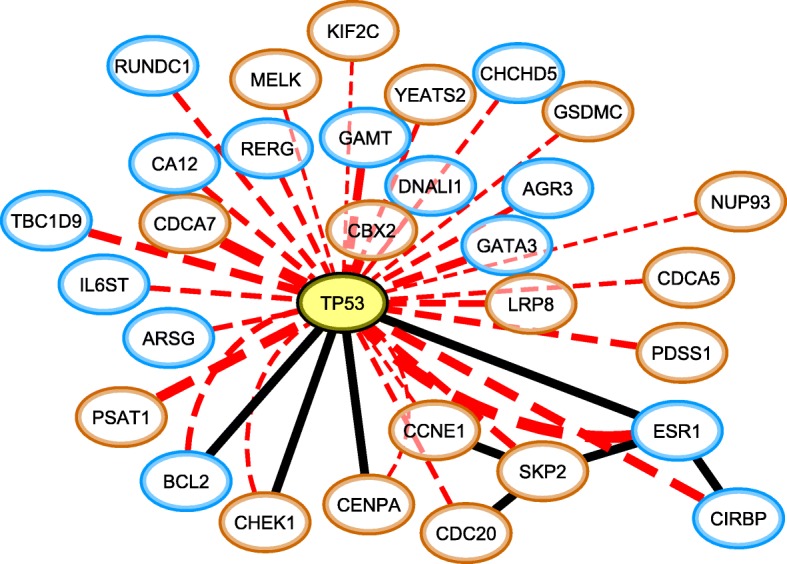
The 30 genes of which the expression regime co-occurs most significantly with mutations in TP53 (indicated in yellow). The red lines connect TP53 to all genes with which it displays a co-occurrence relation, with thickness indicating the association strength (all log_10_(*p*-value) < − 35, far below our conservative lower bound on the significance level). The black lines denote interactions that are present in BioGRID. The expression regimes of the co-occurring genes are indicated with border colors, where orange and blue respectively denote the regimes of high and low expression

As an illustration Fig. 5 graphically shows the 30 genes that co-occur most significantly with mutations in *TP53* in the METABRIC study. The association between *TP53* and *ESR1*, which was found in both datasets, has been reported in literature [41, 42]. Additionally, the presence of a mutation in *TP53* and subsequent loss of activity in *ESR1* is an indicator of a poor prognosis (Additional file 4: Figure S4). Note that from the 30 most significantly co-occurring genes, only 4 are directly interacting with *TP53* according to BioGRID [43, 44]. For instance, *CDCA7* is the second most significantly co-occurring gene, but does not interact with *TP53*. By lowering the threshold on the significance of the association level between *TP53* and expressed genes, an increasing number of genes are added to the subnetwork, revealing that indeed many of the genes are indirectly connected to the mutations in *TP53*. We also observed that out of the 30 genes displayed in Fig. 5, 21 genes have a shortest path distance of 2 to *TP53* on the BioGRID network, while 4 genes have a distance of 3. This shows that the effect of a genomic aberration can propagate far down an interaction network, obviating the detection of such relations by local network methods.

**Fig. 6:**
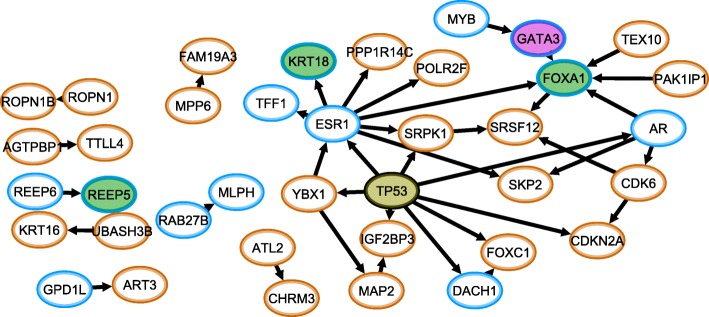
The MLPH-FOXA1 subnetwork identified using the co-occurrence measures between different data sources. We defined a subgroup of samples that were in the low expression regime for both MLPH and FOXA1. Then we calculated all significantly co-occurring expression regimes, mutations and copy number alterations. The expression regimes are denoted by blue (low expression) and orange (high expression) border color. The node color is used to indicate genes that are significantly mutated (yellow), amplified (purple) and deleted (green). Arrows indicate the estimated direction of the association (see text)

Figure 5 illustrates how mutation data can be integrated with expression data. However, it is also possible to integrate all data sources, including the copy number data that is available. We therefore developed a query-based workflow that allows to perform a focused analysis of a particular phenotype, condition or relation that is relevant only to a subgroup of a cohort. As an example, we investigated the *MLPH-FOXA1* relation, that was among the top two most significant expression associations in both TCGA and METABRIC. For all samples with low *MLPH* and low *FOXA1* expression, additional genes with significantly co-occurring expression regimes, copy number changes and/or mutations were identified. The identified genes were mapped on the BioGRID network and the direction of the interaction was deduced using conditional probabilities (see materials and methods). The resulting network is shown in Fig. 6.

The network consists of three hubs (*TP53, EGFR, FOXA1*), but note that *MLPH* is not connected to *FOXA1*. Indeed, this association has not yet been reported in literature. The genomic changes associated with the *MLPH-FOXA1* association, are amplifications in *GATA3* and deletions in *FOXA1*, *KRT18* and *REEP5*, alongside mutations in *TP53*.

In total we identified 113 genes that are strongly associated with *MLPH-FOXA1* (see methods), but only 38 genes could be mapped onto known interactions from BioGRID (Additional file 5: Figure S5). Lowering the significance threshold to include more associations increases the fraction of genes that can be connected. For instance, if we use a conservative lower bound on the significance level (see methods) we find known interactions between 902 out of 1728 associated genes.

Using this workflow we analyzed the top 1000 strongest associations that were found when analyzing the expression data. Interestingly, we found that for the METABRIC study 695 associations co-occurred significantly with mutations or copy number alterations. For TCGA this number was lower, here 365 associations could be linked to an underlying genomic change. These results clearly show that co-occurrence measures on expression regimes indeed allow for the discovery of associations that are condition specific.

## Discussion

In this work a straightforward and intuitive workflow has been proposed to integrate expression data genomic information. The expression data is first discretized into expression regimes, using a GMM clustering based on the BIC criterion. In breast cancer, we observed that important breast cancer genes actually follow a bimodal distribution, such that it suffices to discretize the data into only two expression regimes. This is confirmed when training a Random Forest on the discretized data to predict the PAM50 subtype, resulting in a slightly better classification performance on the binarized data compared to data that allowed for more than two regimes. The binarized model also scored on par with a classifier trained on the continuous data, showing that the binarized data retains important clinical information. This is confirmed when we calculate the hazard ratio for each gene, comparing the survival chances of patients that are in the low expression regime to those in the high expression regime. We find that 4 out of 5 genes have been reported in literature and that the hazard ratios are higher than expected by chance.

By calculating the *p*-value under independence assumption, many significant associations were found in the expression data, showing that this co-occurrence measure can indeed be used to identify important relations from expression data. We compared the *p*-values against two other association measures, the Mutual Information and the Pearson correlation coefficient and compared our findings on the METABRIC study to TCGA-BRCA. In general all measures demonstrated a rather poor overlap, which is presumably caused by the intrinsic differences between the two studies. Nevertheless, we found some important overlap such as the association between *MLPH* and *FOXA1*. In general the association measures seem to prioritize different associations, showing that association studies can greatly benefit from the inclusion of different association measures.

We’ve also demonstrated that the discretized data is consistent with the genomic information, in the sense that many expression regimes co-occur significantly with mutations and copy number changes. Here again, we obtain p-values that are so low that these occurrences are extremely unlikely to have occurred by chance. Remarkably, the two datasets show a better agreement when associating genomic information with expression data. This result aptly illustrates the relevance of data-integration, where in our case genomic information is used to unravel the many signals present in expression data. This increase in consistency is also noted when comparing the top associations found using MI to those found with co-occurrence.

The biggest advantage of using co-occurrence measures such as correlation or MI lies in the fact that the samples for which the association holds are also known. By taking the *MLPH*-*FOXA1* association as an example, a query-based workflow was presented to integrate genomic information such as mutation and copy number data in an interpretable way. The proposed workflow allows to quickly mine associations between different data sources and discover important relations. It is also very flexible, allowing for the integration of expression data with many different data sources, provided that the data is qualitative. However, confronting our findings with known interactions in BioGRID showed that many of these associations cannot yet be explained. Indeed, many of the associations found using the co-occurrence measure are actually strongly related to mutations or copy number changes, such that these some associations might represent cases of genetic rewiring that are not yet well understood.

Our results show that the GMM discretization of expression data is a viable strategy for performing data integration, and our results indicate that the found expression regimes have both a clinical and biological meaning. This illustrates again that there are more reasons than merely mathematical convenience to discretize expression data [9]. Nevertheless, every sample that is mislabeled in this discretization step, is irreversibly lost in downstream analysis. Moreover, it has to be further investigated how different preprocessing of the data influences the quality of the proposed discretization approach. Additional work is also required to improve the consistency between the microarray and RNA-seq data. In this work, the same discretization scheme was applied to both datasets, but a better overlap might be obtained if the data is processed in a more technology-specific manner.

## Materials and methods

### Dataset

The data was taken from the METABRIC study [29, 30] and TCGA-BRCA. METABRIC consists of whole-genome microarray data (1904 samples), whole-genome aCGH data (2173 samples) and targeted mutation data (2394 samples) for a panel of 174 genes. The aCGH data and expression data have been preprocessed as described in Margolin et al. [29]. The aCGH data has hereby been processed to contain 5 discrete values indicating whether many gains/deletions, some gains/deletions or no gains or deletions exist in a sample. The TCGA-BRCA data consists of whole genome RNA-seq data (Illumina HiSeq 2000 RNA Sequencing platform, 1218 samples), copy number data (Affymetrix Genome-Wide Human SNP Array 6.0 platform, 1080 samples) and mutation data (1057 samples). The expression data was normalized using FPKM-UQ and then log-transformed. The copy number data was processed using GISTIC2 [45] from the TCGA FIREHOSE pipeline and binned into the same five categories as the METABRIC study. The somatic mutations were called using MuTect 2 [46]. Table 2 shows the clinical subtypes for both datasets, where the clinical information for TCGA-BRCA was taken from Berger et al. [47]. To visualize the interactions between genes, we used the BioGRID interaction database – version 3.4.161 [43, 44] and Cytoscape [48].

**Table 2:** The percentage of patients in each PAM50 subtype, comparing METABRIC and TCGA-BRCA

Subtype	METABRIC	TCGA-BRCA
LumA	35.46	51.80
LumB	24.06	19.26
Her2	11.35	7.56
claudin-low	11.04	NC
Basal	10.59	17.70

### Preprocessing

For many genes it can be observed that expression follows a bimodal distribution, as is illustrated in Fig. 1 for *ERBB2*. The expression profile can be divided into different ‘regimes’, that can be described by a Gaussian Mixture Model (GMM) [49]. Essentially, the expression profile of each gene is clustered into *n* clusters or regimes, where the Bayesian Information Criterion (BIC) is used to determine the optimal number of clusters [31]. This idea is similar to using k-means for the clustering of expression profiles [9, 50], but here the number of clusters (i.e. regimes) is derived from the data and can thus vary between genes. An important parameter for the discretization is the maximum number of regimes that an expression profile can consist of. For most of the results shown in this work, the maximum number of regimes was set to 2 (see results section). For this value, bimodal expression profiles will be clustered into 2 regimes, and the data is binarized by replacing the continuous expression measurement by a ‘0’ and ‘1’ indicating the cluster or regime the expression measurement belongs to. In total 14,341 out of 24,630 transcripts are bimodal, for the METABRIC study, while 49,184 out of 56,861 transcripts were found to be bimodal in the TCGA study. The copy number data was binned into three categories: deletions, unchanged and amplifications. Between METABRIC and TCGA-BRCA 15864 transcripts had an identical gene symbol, the correlation and unfiltered co-occurrence from Fig. 3a were calculated on this dataset. From these 15,864 genes only 7022 were bimodal in this both datasets. This smaller dataset was used to calculate the MI filtered and co-occurrence filtered.

### Clinical information

To check that important breast cancer genes have a multimodal expression profile, we downloaded the KEGG breast cancer pathway, which consists of 15 highly curated genes related to breast cancer [32]. We only considered the genes that were over- or underexpressed or amplified/deleted, resulting in a list of ten genes (*ESR1*, *FGFR1*, *CCND1*, *EGFR*, *KIT*, *Notch1*, *Notch4*, *FZD7*, *LRP6, ERBB2*).

To calculate the hazard ratios we used the lifelines module [51] in Python to fit a Cox proportional hazard model to the patient survival data, for each gene. To correct for patient age the model had two covariates, patient age and a binary variable indicating the regime of the gene. The hazard ratio for each gene was then calculated as max(exp(*γ*), exp(−*γ*)), where *γ* is the coefficient belonging to the binary variable in the Cox proportional hazard model. The resulting ratio represents the hazard ratio for the regime with the poorest prognosis and is always larger than 1. Hazard ratios were only calculated when both regimes of a gene occurred in at least 10% of the samples, this to assure that the obtained ratios were representative. To obtain the random hazard ratios, we permuted the samples and calculated the hazard ratio in the same way.

### Co-occurrence measures

To analyze the relations between genes two different measures were used that define how closely two genes are related, conditional on their status (which can be an expression or mutational status). A first measure can be interpreted as the *p*-value of co-occurrence under independence assumption. The second measure is an estimate of the conditional probability that a gene will be in a certain regime, given the regime of another gene. Both measures will be illustrated using the expression status of the genes involved in the association.

The first measure is a p-value under the hypothesis that two genes are independent, i.e. the expression regime of one gene is independent from the other. Under this independence assumption, we can approximate the probability that gene A is in regime i while gene B is in regime j:

$$ {\displaystyle \begin{array}{r}P\left({A}_i\cap {B}_j|{H}_0\right)=P\left({A}_i\right)P\left({B}_j\right)\\ {}\approx \frac{\mid {A}_i\mid \mid {B}_j\mid }{N_{samples}^2}\end{array}} $$

The expected number of co-occurrences is compared to the observed number, under a binomial distribution. This work focuses on finding regimes that co-occur more than expected by chance, as such one sided testing suffices. The resulting quantity can be interpreted as a p-value under the independence assumption, i.e. a measure that expresses how much more the expression regimes of two genes co-occur than expected by chance.

The second measure can be interpreted as the conditional probability that a gene will be in a certain expression regime, given the regime of another gene. Using the same example as above, we have:

$$ {\displaystyle \begin{array}{r}P\left({A}_i|{B}_j\right)=\frac{P\left({A}_i\cap {B}_j\right)}{P\left({B}_j\right)}\\ {}\approx \frac{\mid {A}_i\mid \cap \mid {B}_j\mid }{\mid {B}_j\mid}\end{array}} $$

With |*A*_*i*_| ∩ |*B*_*j*_| the number of samples in which gene A is in regime ‘i’ and gene B in regime ‘j’, i.e. the number of times *A*_*i*_ and *B*_*j*_ co-occur. Remark, that the conditional probability has the undesirable property that it can still be close to one, when both ∣*A*_*i*_ | and ∣*B*_*j*_∣ are small. This makes it unsuited for identifying associations in large datasets. However, as the conditional probability is a directed measure, i.e. in general *P*(*A*_*i*_| *B*_*j*_) ≠ *P*(*B*_*j*_| *A*_*i*_), it can be used to deduce a direction between associated genes (cf. Figure 6).

The measures can be extended in a trivial way to assess relations between genes that involve a genomic status (mutation, amplification, deletion) e.g. by counting the number of times an aberration co-occurs in both genes or by counting the number of times an aberration in one gene co-occurs with a specific expression regime of another gene.

### Mutual information (MI)

Mutual information is an association measure that can is often used in the field of information theory to express the dependence of two random variables [26, 52]. In the continuous case, the MI between two random variables X and Y is defined as:

$$ MI\left(X,Y\right)=\iint P\left(x,y\right)\ \log \left(\frac{P\left(x,y\right)}{P(x)P(y)}\right) dx\  dy $$

In our case X and Y both correspond to the expression measurements of a gene in different samples. Because the underlying distributions p(x), p(y) and p(x, y) are unknown, the MI cannot readily be calculated. However, there exist methods to estimate the MI directly from the continuous expression measurements [53]. In this work, however, we calculate the MI between the discretized expression profiles of gene A and gene B as:

$$ {\displaystyle \begin{array}{r} MI\left(A,B\right)=\sum \limits_i\sum \limits_jP\left({A}_i\cap {B}_j\right)\log \left(\frac{P\left({A}_i\cap {B}_j\right)}{P\left({A}_i\right)P\left({B}_j\right)}\right)\\ {}=\sum \limits_i^{\mid X\mid}\sum \limits_j^{\mid Y\mid}\frac{\mid {A}_i\cap {B}_j\mid }{{\mathrm{N}}_{samples}}\log \left(\frac{\mid {A}_i\cap {B}_j\mid {N}_{samples}}{\mid {A}_i\mid \mid {B}_j\mid}\right)\end{array}} $$

Where we see that the MI is actually closely related to the concept of co-occurrence from the previous paragraph. Indeed, each term in the summation is a co-occurrence measure, as it described how well regime *A*_*i*_ overlaps with regime *B*_*j*_. The big difference is that MI quantifies the association between two genes based on the sum over all possible regimes, whereas a co-occurrence measure only depends on one regime per gene.

### Correction for multiple hypothesis testing

Using the *p*-value under independence as an association measure has the advantage that the measure has a clear interpretation and that all pairs at a given significance level can be determined. However, because of multiple hypothesis testing, correctly determining the significance level is by no means trivial. A common approach to deal with this problem is to rely on the False Discovery Rate (FDR) [54, 55], as classical measures such as Bonferroni are deemed too conservative [56]. However, the scope of this work is not to present an extensive list of significant associations, and we leave the significance level as a model parameter such that the user can decide. Whenever we mention the number of significant pairs, we use the Bonferroni correction as a conservative lower bound. Aiming for a significance level *α*, the corrected threshold θ for all associations between two datasets is computed as:

$$ \vartheta =\frac{\alpha }{N\ {n}_{reg}\ M\ {m}_{reg}\ } $$

Where *n*_*reg*_ and N are the number of regimes and number of genes in the first dataset and *m*_*reg*_ and *M* that for the second dataset. In this work we set *α* = 0.001, i.e. we work at a 0.001 significance level, which for the METABRIC expression dataset results in a threshold of $\frac{0.001}{{\left(14341\times 2\right)}^2}\approx 1.26\times {10}^{-12}$.

### Data integration using co-occurrence

To create Fig. 5 and Additional file 5: Figure S5, all expression regimes, mutations, deletions and amplifications that significantly co-occur with the subgroup of samples that have a low expression in *MLPH* and *FOXA1* are calculated. To keep the size of the network small enough for displaying it, only expression regimes with a *p*-value < 10^− 50^ were kept (154 genes in total). For the copy number and mutations a threshold p-value < 10^− 20^ was taken, retaining only *TP53* as significant mutation, 2243 deletions and 390 amplifications. All expression regimes that significantly co-occurred with the *TP53-ESR1* relation were mapped onto the BioGRID network [43, 44], retaining 38 interactions between only 38 of the 113 genes. All links were filtered by imposing that between every link A_i_ – B_j_, either P(A_i_| B_j_) > 0.5 or P(B_j_|A_i_) > 0.5 for their relevant regimes, as not all genes that co-occur with *MLPH*-*FOXA1* are necessarily co-occurring with each other. However, for these parameter settings all found interactions passed the filtering criterion. The direction from the interaction was deduced from the expression regimes using the simple heuristic that if P(A_i_| B_j_) > P(B_j_ |A_i_), then B_j_ → A_i_. For copy numbers changes, it was verified that the changes co-occurred significantly with the expression regimes in the same gene, using the same threshold that was used to select the copy numbers (p-value < 10^− 20^). Finally, all genes and their attributes (i.e. genomic status) were visualized using Cytoscape [48].

## Reviewers’ comments

### Reviewer’s report 1: Dirk Walther

Review comment: My two main questions/ concerns relate to the statistical methodology: When establishing correlations between discrete variables, the first metric that comes to (my) mind is mutual information (MI) - or something similar (Jaccard distance). In fact, MI has been used to correlate genes before (Steuer et al. 2002, Bioinformatics, “The mutual information: …” - I think, you should also cite this paper). Instead, binomial testing of co-occurrence of states is used. However, there are four possible states for two-state variables (++,--,+−,−+), which need to be considered. But they are not independent. So why not used MI, which does everything in one go and automatically accounts for number of different states per gene?

*Author’s response: We would like to thank the reviewer for this important point. Indeed, Mutual information would be the first choice for the discretized data and we’ve added a comparison between mutual information and the binomial testing procedure. The results seem to indicate that these two associations measures find (or at least rank) different associations. This is indeed due to the fact that the Mutual information considers all states of a gene at once, which make MI a robust and reliable association measure. However, because MI is calculated over all states at once (and thus all samples), the found associations can no longer be related to a subgroup of samples. This is different for the proposed co-occurrence measures. For instance, in the case of the MLPH-FOXA1 association that we found, we know that this association is present in all samples where both MLPH and FOXA1 are in a low regime (information that is lost in MI by aggregating over all the states). This allows us to obtain not only the association, but also the subgroup of patients in which the association is present. Because, we exactly know in which samples the association MLPH-FOXA1 is present we can again calculate the co-occurrence between this associations and expression regimes/mutation data/copy number data of other genes. We’ve used this simple concept to perform data integration.*

Review comment: You can also establish significance by using randomized data. - Multiple testing correction: 142,827 gene pairs with co-occurring regimes with *p* < 1E-30, are being reported. The authors do not mention any performed testing for multiple testing. This MUST be done. And if not done so, authors should introduce proper correction. Actually, throughout the manuscript. Though, one way to do it, is to compare to random data as done by the authors in the paragraph above (91 vs. none).

*Author’s response: Another very important remark. We’ve added some more text to better justify the significance levels that were chosen throughout the work. When seen as an association measure, the p-values represent a number that indicate how strong the association between two genes is. In that case, selecting a significance level is the same as deciding upon a threshold for the correlation coefficient to determine which pairs are correlated and which aren’t. However, because the associations have this interpretation of being a p-value, there exist statistically sound ways to determine a threshold, finding a delicate balance between sensitivity and specificity, such as FDR. The fact that this interpretation exists could be considered an advantage of the co-occurrence over other association measures such as correlation or MI. We’ve considered and tried random data for testing, but it turns out to be very hard to accurately estimate the tail of the distribution. However, the results obtained with this random testing procedure are in line with the conservative lower bound used throughout the work.*

Review comment: The GMM approach should be explained in more detail. Perhaps, it would even make sense to treat it as part of results. When reading the article start-to-finish, it is not clear upon first encounter (l106), what GMM actually is and that, in fact, it is the heart of the study.

*Author’s response: We completely agree with the comment and have modified the manuscript such that this should be more clear to the reader.*

Review comment: Please provide some overview statistic: for the dataset(s) used, how many genes were found to be unimodal, bimodal, > 2 states etc.. The used experimental dataset should be explained more. How many different subtypes have been described (is 5 (l264) the relevant number? unclear).

*Author’s response: We have included additional information on this in the methods section.*

Review comment: The term “regime” is a bit nebulous. At least the authors should provide a sentence or two as to what they if in mind when talking about regimes.

*Author’s response: Again a very valid comment. We have added additional clarification in results section.*

Review comment: Frequently, the term “binarization” is used. Even though the methodology would also allow for more than two states. Either the authors mean “binning” or indeed, a two-state (binary) situation. Please explain/ make unambiguous.

*Author’s response: We would like to thank the reviewer for the many relevant comments, which will really improve the quality of this work. Indeed, the proposed workflow can be extended to the general case of n regimes. There is one slight caveat, as a high number of regimes might imply that some regimes contain a low number of samples. For these regimes it will be impossible to achieve high p-values. As a part of our results we show that working with 2 regimes,* i.e. *binarizing the expression data, is actually sufficient to recover many of the signals in the data. We have elaborated these results a bit more in the results section.*

### Reviewer’s report 2: Francisco Garcia

Review comment: Is the code available in any repository? Reproducibility is a good and necessary value.

*Author’s response: The code will soon be made available on Github. Everything is written in Python and builds upon pandas and numpy.*

Review comment: Did you try your strategy in more real or simulated datasets?

*Author’s response: We’ve added a comparative analysis, that validates our results on another large breast cancer dataset (TCGA-BRCA). Our results probably underestimate the overlap between the datasets, as we didn’t take any platform bias into account (METABRIC uses microarray expression and TCGA RNA-seq). Nevertheless, we observe that significant pairs found on METABRIC can often be recovered in the TCGA dataset.*

Review comment: Did you compare your results with other methods for the same dataset? Maybe it would be a good proof to demonstrate the power of this new approach.

*Author’s response: This is a very good point, and something that was indeed missing in the first version of this work. In the new version we’ve compared the co-occurrence measures to two other measures, Pearson correlation coefficient and Mutual Information.*

### Reviewer’s report 3: Isabel Nepomuceno

Review comment: Authors propose a straightforward and intuitive workflow to integrate genomic information with expression data. Furthermore, they claim that they developed a query-based workflow that allows performing a focused analysis of a particular phenotype, condition or relation that is relevant to a subgroup of the samples. For this assertion, I expected a stand-alone software or web to reproduce the analysis. Authors should provide at least the script to reproduce the study and explain the tools used to implement it.

*Author’s response: The Python code for performing the analysis will be made available on Github, such that people can reproduce our results and run their own analyses.*

Review comment: Authors claim that co-expression measures might not be the measure of choice if the expression of two genes is related only under a specific set of conditions. In this case, it should be mentioned some methods based on local search strategy, which try to extract the similarities under a subset of samples using biclustering as [1] or other methods that partition the search space as [2]. ( [1] Mitra, Sushmita, et al. “Gene interaction–An evolutionary biclustering approach.” Information Fusion 10.3 (2009): 242–249. [2] Nepomuceno-Chamorro, Isabel A., Jesus S. Aguilar-Ruiz, and Jose C. Riquelme. “Inferring gene regression networks with model trees.” BMC bioinformatics 11.1 (2010): 517).

*Author’s response: We thank the reviewer for pointing out the missing references to the local search methods. Indeed, the work we present is very much related to the concept of bi-clustering, but the bi-clustering methods and other local search approaches were not properly referred to, this is now added.*

Review comment: General structure of the paper is clear, but there are several confusing details. I endorse the publication, but I strongly suggest the authors to revise next comments: In subsection “Clinical relevance of the expression regimes” the PGAP3 gene is ranked on the 10th place in the list, do the authors refer to the list of genes in Table 1? This list only shows the first 5 genes. - In Table 1, the hazard ratio is shown, how is it calculated? This is not mentioned on the section materials and methods. Figure references should be checked carefully. On page 3 first paragraph authors mentioned that Fig. 2 shows a boxplot, but the boxplot is Fig. 1. On page 4, the Fig. 3b is referenced instead of Fig. 2b, I guess. And in the last paragraph of this section authors claim that “the resulting network is shown in Fig. 5” and this figure shows a distribution of regimes. Throughout the results section it is not clear which co-occurrence measure is used from the two explained in the subsection “Co-occurrence measures”. I guess it is both of them, but it is confusing when it is used one or the other. The datasets used are not explained in detail. The microarray is described by the number of samples, but the number of attributes and the number of subtypes are not mentioned. The equations of the two co-occurrence measures use the intersection symbol instead of the logical operator conjunction “and”.

*Author’s response: We have revised these comments and would like to thank the reviewer for pointing them out.*

## Additional files

Additional file 1: **Figure S1.** Overlap between the 1000 strongest associations found in the METABRIC expression data using different association measures. The largest overlap can be observed between both discrete measures (Mutual Information and Co-occurrence). (PDF 12 kb)

Additional file 2: **Figure S2.** the subnetworks that are present in Additional file 6: Table S1, where the red dotted lines connect significantly co-occurring genes pairs. The expression regime of the gene is indicated with a blue and orange border color, for low and high expression respectively. Remark that for every subnetwork, the corresponding samples are known such that each subnetwork corresponds to a small bi-cluster. (PDF 3 kb)

Additional file 3: **Figure S3.** Fraction of associations from TCGA-BRCA that are found back in METABRIC for different significance levels. (PDF 29 kb)

Additional file 4: **Figure S4.** Survival characteristics of the patients that have a mutation in TP53 and low expression in ESR1 (TP53-ESR1, green). The survival curve is compared against a group that has no mutation in TP53 and high expression in ESR1 (Baseline, blue), a group that has only mutations in TP53 but no low expression in ESR1 (only TP53, orange), and a group that has only low expression in ESR1 (only ESR1, red). It can be observed that low expression in ESR1 is associated with a poor prognosis, irrespective of the mutation status of TP53, but that the co-occurrence of a mutation in TP53 and low expression in ESR1 seems to be less aggressive compared to the independent occurrence of either. (PDF 24 kb)

Additional file 5: **Figure S5.** all 154 genes that are found to be co-occurring with the MLPH-FOXA1 association, where the red dotted lines connect all genes with a clinical subgroup or phenotype of interest (in this example the subgroup corresponds to all patients that have are in the low expression regime of both MLPH and FOXA1). The black edges correspond to interactions that are found in BioGrid and represent the subnetwork that is depicted in Fig. 6. The arrows indicate the estimated direction of the interaction. For each gene, the expression regime that is found to significantly co-occur with the MLPH-FOXA1 association is indicated with an orange (high expression regime) or a blue (low expression regime) border color. Colors are used to indicate mutations (yellow), amplifications (purple) and deletions (green) that co-occur significantly with the MLPH-FOXA1 relation. (PDF 22 kb)

Additional file 6: **Table S1.** The thirty most co-occurring expression regimes. (DOCX 18 kb)
