# Supplementary figures and images for: Associating expression and genomic data using co-occurrence measures
Source: Biol Direct. 2019 May 9;14:10. doi: 10.1186/s13062-019-0240-2 (PMC6507230; doi:10.1186/s13062-019-0240-2)

Correlation

Co-occurrence

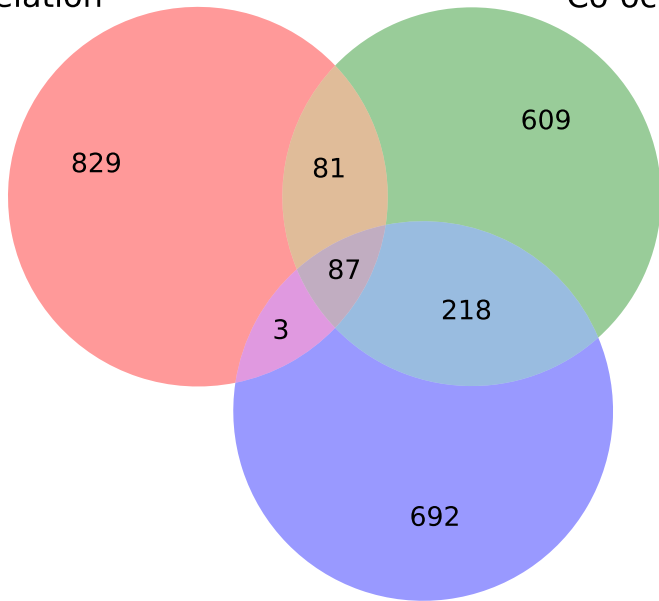

MI filtered

Supplement: Supplementary file 1 — Figure S1. Overlap between the 1000 strongest associations found in the METABRIC expression data using different association measures. The largest overlap can be observed between both discrete measures (Mutual Information and Co-occurrence). (PDF 12 kb) [file 13062_2019_240_MOESM1_ESM.pdf]

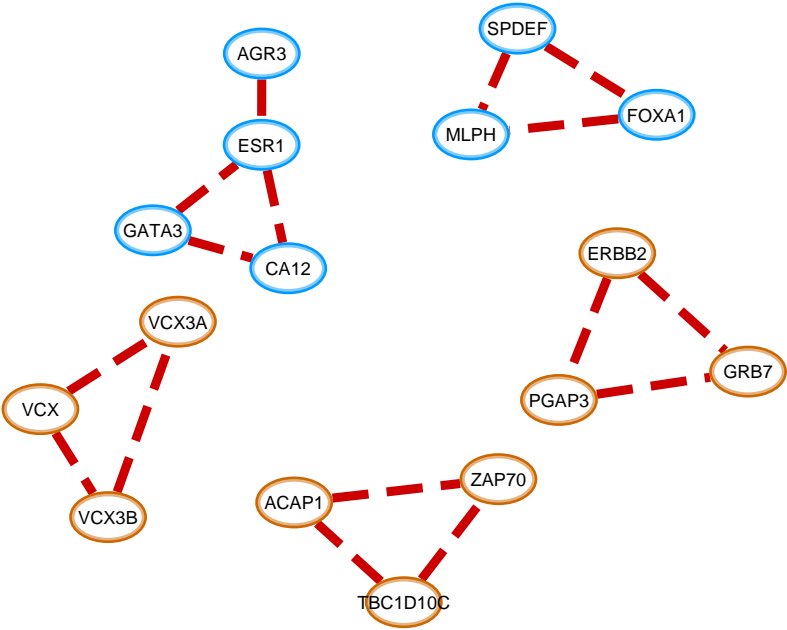

Supplement: Supplementary file 2 — Figure S2. the subnetworks that are present in Additional file 6: Table S1, where the red dotted lines connect significantly co-occurring genes pairs. The expression regime of the gene is indicated with a blue and orange border color, for low and high expression respectively. Remark that for every subnetwork, the corresponding samples are known such that each subnetwork corresponds to a small bi-cluster. (PDF 3 kb) [file 13062_2019_240_MOESM2_ESM.pdf]

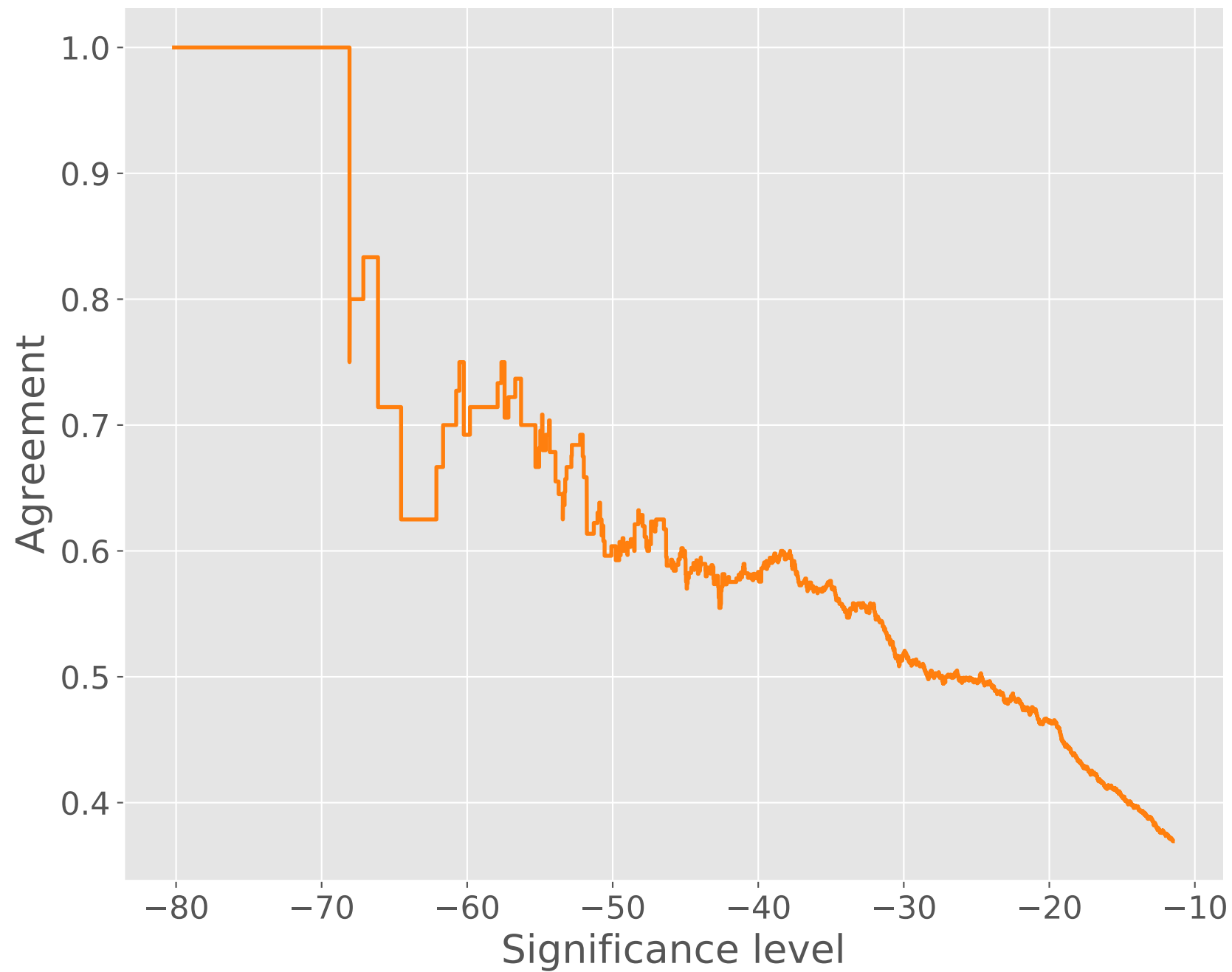

Supplement: Supplementary file 3 — Figure S3. Fraction of associations from TCGA-BRCA that are found back in METABRIC for different significance levels. (PDF 29 kb) [file 13062_2019_240_MOESM3_ESM.pdf]

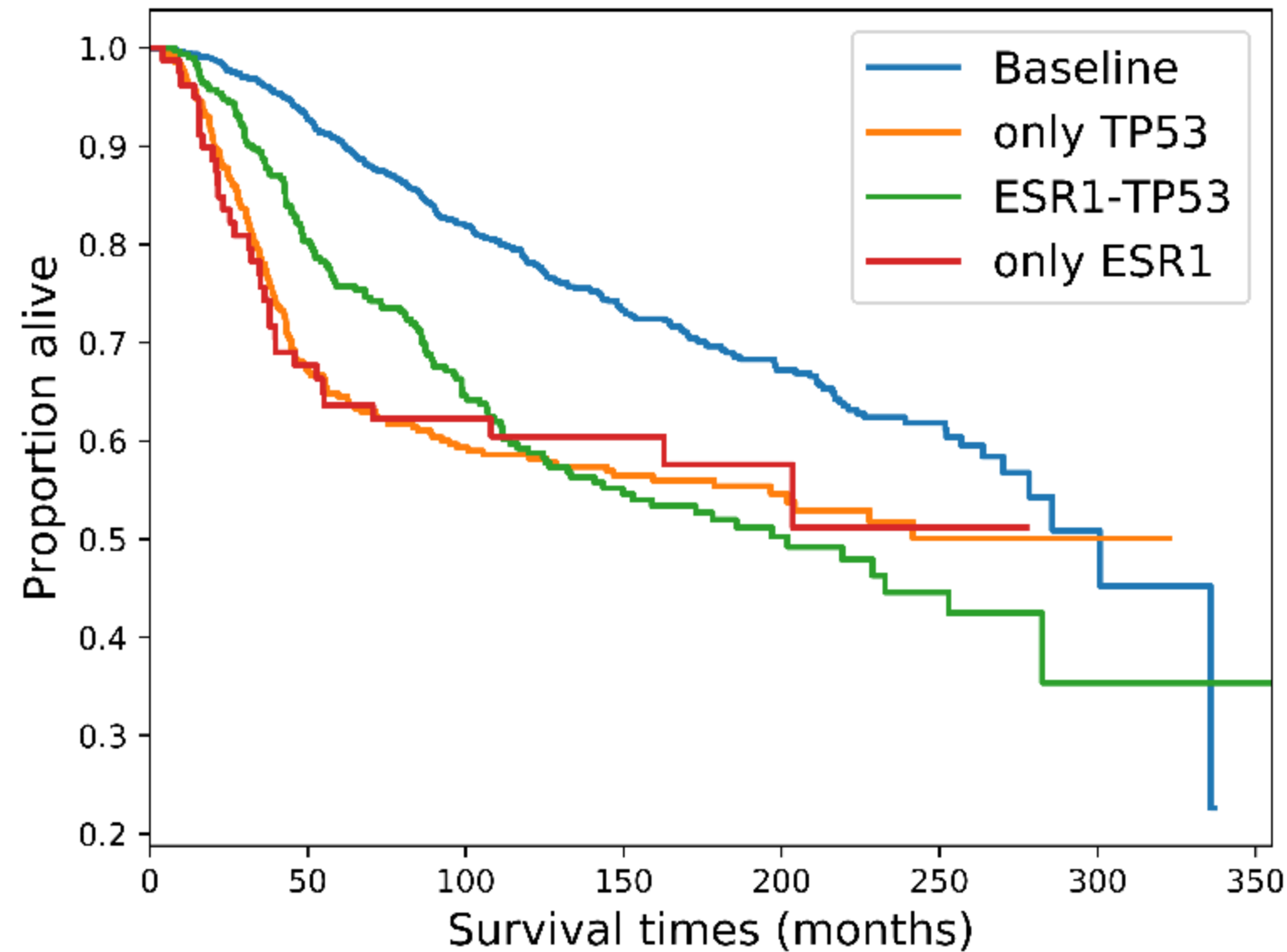

Supplement: Supplementary file 4 — Figure S4. Survival characteristics of the patients that have a mutation in TP53 and low expression in ESR1 (TP53-ESR1, green). The survival curve is compared against a group that has no mutation in TP53 and high expression in ESR1 (Baseline, blue), a group that has only mutations in TP53 but no low expression in ESR1 (only TP53, orange), and a group that has only low expression in ESR1 (only ESR1, red). It can be observed that low expression in ESR1 is associated with a poor prognosis, irrespective of the mutation status of TP53, but that the co-occurrence of a mutation in TP53 and low expression in ESR1 seems to be less aggressive compared to the independent occurrence of either. (PDF 24 kb) [file 13062_2019_240_MOESM4_ESM.pdf]

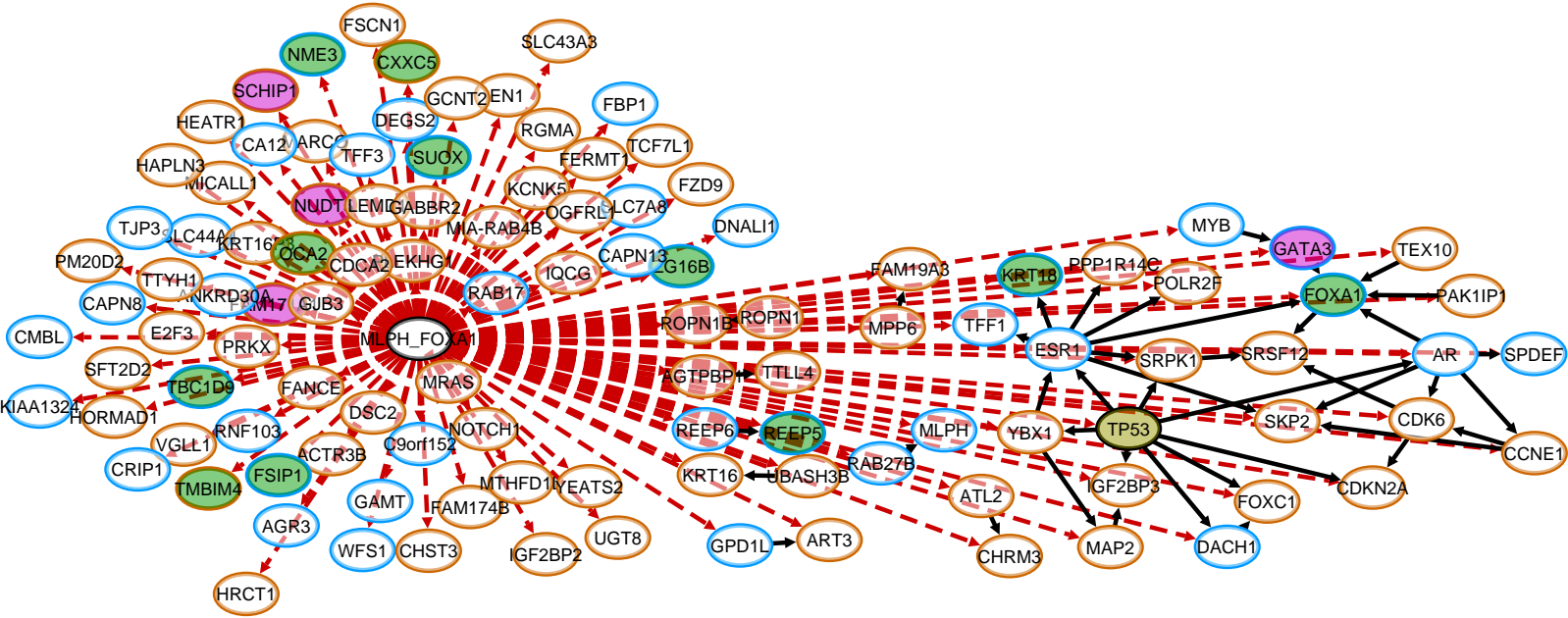

Supplement: Supplementary file 5 — Figure S5. all 154 genes that are found to be co-occurring with the MLPH-FOXA1 association, where the red dotted lines connect all genes with a clinical subgroup or phenotype of interest (in this example the subgroup corresponds to all patients that have are in the low expression regime of both MLPH and FOXA1). The black edges correspond to interactions that are found in BioGrid and represent the subnetwork that is depicted in Fig. 6. The arrows indicate the estimated direction of the interaction. For each gene, the expression regime that is found to significantly co-occur with the MLPH-FOXA1 association is indicated with an orange (high expression regime) or a blue (low expression regime) border color. Colors are used to indicate mutations (yellow), amplifications (purple) and deletions (green) that co-occur significantly with the MLPH-FOXA1 relation. (PDF 22 kb) [file 13062_2019_240_MOESM5_ESM.pdf]
