# Supplementary material for: Associating expression and genomic data using co-occurrence measures
Source: Biol Direct. 2019 May 9;14:10. doi: 10.1186/s13062-019-0240-2 (PMC6507230; doi:10.1186/s13062-019-0240-2)
Supplement: Supplementary file 6 — Table S1. The thirty most co-occurring expression regimes. (DOCX 18 kb) [file 13062_2019_240_MOESM6_ESM.docx]

*Supplemental table 1: the thirty most co-occurring expression regimes.*

| Gene A | Gene B | p-value  (log_10_) | Count | In BioGRID |
| --- | --- | --- | --- | --- |
| ROPN1B 1 | ROPN1 1 | -132,54 | 399 | True |
| FOXA1 0 | MLPH 0 | -115,90 | 245 | False |
| MAFA 1 | CDA 1 | -115,43 | 611 | False |
| OR2B3 1 | LOC100289058 1 | -115,11 | 275 | False |
| S100A9 1 | S100A8 1 | -112,14 | 508 | True |
| ESR1 0 | GATA3 0 | -111,63 | 384 | False |
| UGT2B11 1 | UGT2B28 1 | -110,04 | 585 | False |
| ICOS 1 | CTLA4 1 | -109,65 | 508 | False |
| FOXA1 0 | SPDEF 0 | -109,63 | 215 | False |
| ZAP70 1 | ACAP1 1 | -109,15 | 384 | False |
| CA429430 1 | OR2B3 1 | -107,97 | 278 | False |
| ERBB2 1 | GRB7 1 | -107,30 | 210 | True |
| LOC100130000 1 | AX746564 1 | -106,82 | 506 | False |
| CA429430 1 | LOC100289058 1 | -106,75 | 282 | False |
| PGAP3 1 | GRB7 1 | -106,28 | 216 | False |
| PGAP3 1 | ERBB2 1 | -106,08 | 203 | False |
| BI047056 1 | OR2B3 1 | -105,24 | 271 | False |
| CA12 0 | ESR1 0 | -105,20 | 370 | False |
| ESR1 0 | AGR3 0 | -105,07 | 377 | False |
| HLA-DRA 0 | HLA-DPA1 0 | -104,39 | 446 | False |
| IQCF5 1 | OR2B3 1 | -104,08 | 271 | False |
| VCX3A 1 | VCX 1 | -103,06 | 183 | False |
| BF434110 1 | DR731427 1 | -102,47 | 333 | False |
| WAS 1 | ARHGAP9 1 | -102,44 | 424 | False |
| IQCF5 1 | CA429430 1 | -102,38 | 287 | False |
| TBC1D10C 1 | ACAP1 1 | -102,33 | 417 | False |
| OR2B3 1 | DR731427 1 | -102,27 | 279 | False |
| IQCF5 1 | LOC100289058 1 | -102,20 | 274 | False |
| VCX3A 1 | VCX3B 1 | -102,12 | 191 | False |
| CA12 0 | GATA3 0 | -102,03 | 362 | False |
